# A case of misdiagnosed leiomyoma of the vulva: A case report

**DOI:** 10.1097/MD.0000000000032868

**Published:** 2023-02-10

**Authors:** Jing He, Wenhua Liu, Xiaoyu Wu, Dingheng Li, Yuanwei Liu

**Affiliations:** a Department of Obstetrics and Gynecology, Hangzhou Women’s Hospital (Hangzhou Maternity and Child Health Care Hospital), Hangzhou, China.; b Department of Obstetrics and Gynecology, Zhejiang Chinese Medical University, Hangzhou, China.

**Keywords:** vulvar leiomyoma, vaginal wall mass, Bartholin gland, case report

## Abstract

Leiomyoma of the vulva is a rare, benign mass that is present on the vulva. Most commonly, they are described as painless, well-circumscribed, solitary tumors that are misdiagnosed as Bartholin cysts before surgery.

**Patient concerns:**

A 45-year-old woman presented with a case of vulvar leiomyoma misdiagnosed as Bartholin cyst preoperatively. A solitary swelling mass measuring 3 cm × 2 cm was found in the left labia majora at the Bartholin gland site on physical examination.

**Diagnoses:**

A vulvar mass extent and vascularity may be determined by imaging. A color doppler flow imaging of the posterior vaginal wall revealed abundant blood flow.

**Intervention:**

To confirm vulvar leiomyoma, surgery and histopathology were performed.

**Outcome:**

After 2 months of follow-up, there were no signs of recurrence in the patient.

**Lessons:**

Rare vulvar leiomyomas are often mistaken for Bartholin’s cysts. It is also difficult to distinguish benign from malignant forms, making vulvar leiomyoma a difficult diagnosis. As there are a few techniques used to differentiate between the nature of the tumor, excisional biopsy seems to be the best current procedure employed in addition to being the treatment of choice for such tumors.

## 1. Introduction

Vulvar leiomyoma is a rare and benign mass on the vulva.^[1]^ Leiomyomas of the vulva are nonspecific and camouflaging. Vulvar leiomyomas are often painless, solitary, and well-circumscribed, however, they are sometimes preoperatively misinterpreted as Bartholin cysts, abscesses, or other benign disorders.^[2,3]^

We discuss the case of a 45-year-old woman who had a vulvar leiomyoma that was initially misdiagnosed as a Bartholin cyst.

## 2. Case report

**Figure 1.**
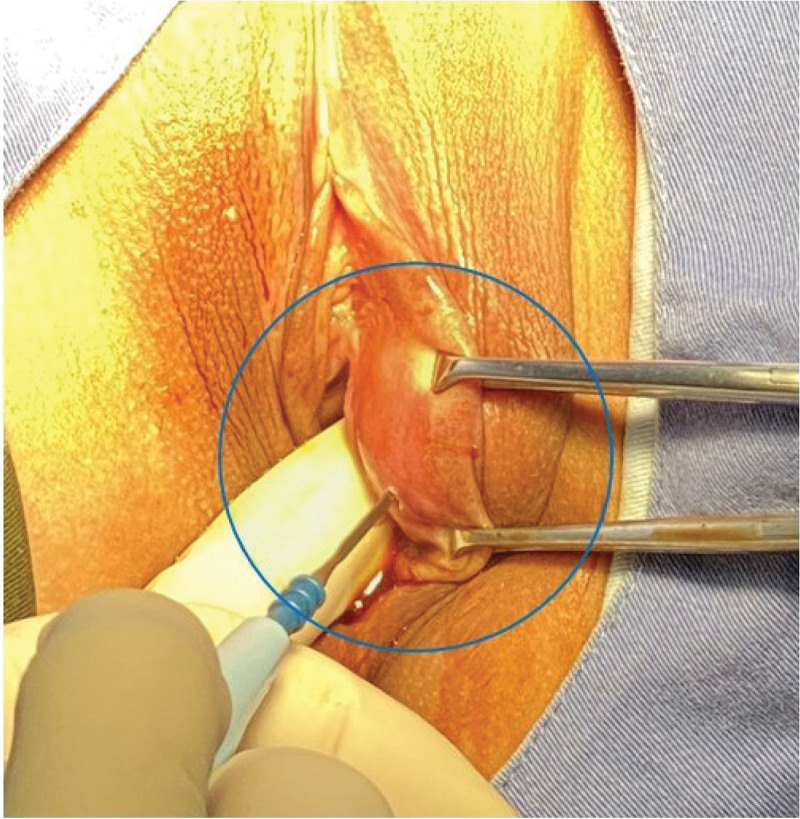
Clinical presentation before surgery of a vulvar leiomyoma (blue circle) found beneath the right labia minora.

**Figure 2.**
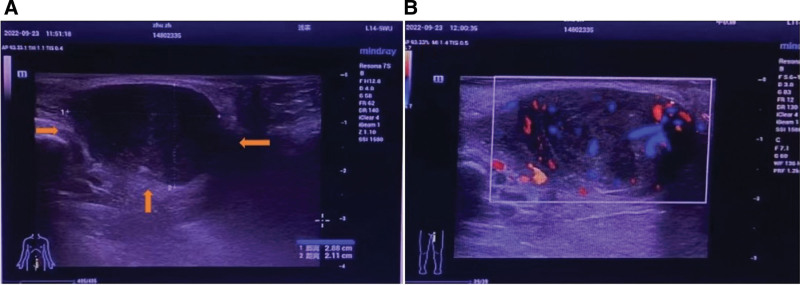
Ultrasound showed a solid mass in the posterior vaginal wall measuring 2.88 × 2.11 cm (A), Color Doppler ultrasound image showing the border (yellow arrows), and vascular nature of vulvar leiomyoma (B).

A 45-year-old female, gravida 1, para 1, resented to the clinic with a 1-year history of left labial mass. There was no history of discharge, fever, weight loss, or a history of malignancy in the family. General examination was unremarkable except for a soft mass that measured 3 × 2 cm in the left labial area (Fig. 1). The mass was medial to the left labia minora. The mass was initially diagnosed as a Bartholin’s cyst. Ultrasonography showed a solid mass in the posterior vaginal wall and copious blood flow by color Doppler flow imaging (Fig. 2A, B).

**Figure 3.**
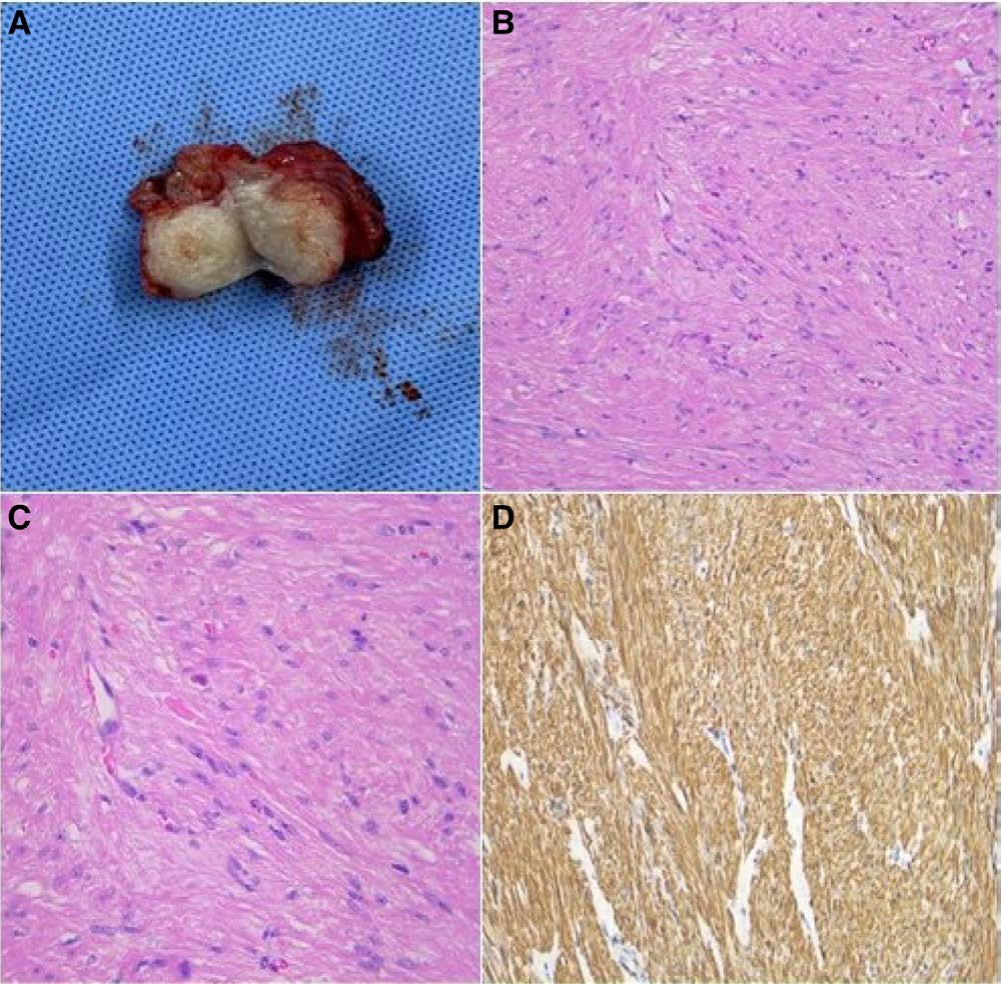
The excised lesion e showed a myoma-like appearance (white whorled cut surface). (A) Histology of the sample. Moderate cellular proliferation of epithelioid cells grouped in fascicles and hazy nests, with certain regions displaying a silk-like appearance (magnification: 200x) (B). With almost no mitotic activity or nuclear pleomorphisms, cells feature bland, homogeneous, round to oval nuclei surrounded by ill-defined eosinophilic cytoplasm (magnification:400x) (C). The smooth muscle was detected by staining with desmin (magnification: 200x) (D).

Due to the tissue’s vascular character, a tiny needle biopsy was not contemplated. The lesion was excised from the patient. The incision at the mucocutaneous junction revealed a 3 × 2 cm soft, meaty, and well-defined mass. The mass was enucleated in fragments and sent to the histopathology lab for analysis. The mass displayed myoma-like characteristics (white whorled cut surface) (Fig. 3A). Histopathology revealed a well-defined nodule with myxoid stroma and spindle cells organized in a spiral. (Fig. 3B). The lesion consists of fascicles of interlacing smooth muscle cells with cigar-shaped nuclei. (Fig. 3C), and smooth muscle was detected by staining with desmin (Fig. 3D). Final analysis revealed a benign vulvar myoma. The patient recovered well after the operation. Throughout the 2-month follow-up, there was no recurrence.

## 3. Discussion

A leiomyoma is a benign tumor caused by smooth muscle cells in any organ.^[4]^ Vulvar Leiomyoma is rare. It comes from smooth muscle cells, spindle cells, and epithelioid cancer cells with eosinophilic cytoplasm, among other things.^[5]^ A vulvar myoma can appear at any age, and until the tumor is surgically removed and microscopic and immunohistochemical tests are performed, the condition is frequently misdiagnosed.

Vulvar leiomyoma is frequently mistaken as Bartholin’s gland cyst, the most common preoperative diagnosis, because the 2 conditions share some of the same presenting symptoms, such as a painless lump and swelling in the area.^[2]^ In the current incident, a clinically verified Bartholin cyst was also present. Bartholin’s cyst and vulvar leiomyoma can be differentiated by the direction and consistency of the labia minora. If the labia minora are everted and the cyst is soft, it is a Bartholin cyst; if they are inverted and the cyst is hard, it is a vulvar leiomyoma. Vulval leiomyoma can also be detected in the clitoris or concealed by another condition.^[6]^

Using magnetic resonance imaging and transvaginal ultrasound, it is possible to diagnose vulvar leiomyoma and distinguish it from leiomyosarcoma.^[7]^ Excision by the surgeon is the treatment of choice.

Many vulval lesions look identical, making it difficult to tell benign from malignant lesions only by looking at them, so diagnosing smooth muscle tumors of the vulva can be difficult. A vulvar leiomyosarcoma may be suggested if 3 of the following criteria are met: the size of 5 cm or more, infiltrative margins, 5 or more mitotic figures per 10 high power fields, and grade 2 to 3 atypia.^[8]^ Regarding their view, our case lacked any of the aforementioned characteristics that suggested a benign leiomyoma diagnosis. Leiomyoma of the vulva should not be considered benign unless a histological study has been performed. Out of Nielsen et al 25 cases of vulval leiomyomas, 4 and 5 were found atypical or sarcomas, respectively.^[8]^ It is recommended, therefore, to surgically remove any vulval tumor for proper histological and immunological evaluation.

## 4. Conclusions

Leiomyoma of the vulvar is an uncommon tumor that is sometimes misinterpreted as Bartholin’s cyst. Leiomyoma of the vulvar is extremely difficult to diagnose since it is difficult to distinguish benign from malignant types. An unusual clinical presentation should prompt ultrasonography and/or magnetic resonance imaging to rule out malignancy, followed by surgical removal of the mass with a safety margin of normal tissue for histopathology and immunohistochemistry.

## Acknowledgments

The authors express their gratitude to the patient who made this work possible, as well as the professionals and researchers that participated in this study. A patient’s informed consent was acquired.

## Author contributions

**Conceptualization:** Jing He.

**Data curation:** Jing He.

**Investigation:** Wenhua Liu.

**Methodology:** Wenhua Liu.

**Project administration:** Wenhua Liu.

**Resources:** Wenhua Liu.

**Software:** Xiaoyu Wu.

**Supervision:** Xiaoyu Wu, Dingheng Li.

**Validation:** Dingheng Li.

**Visualization:** Dingheng Li.

**Writing – original draft:** Yuanwei Liu.

**Writing – review & editing:** Yuanwei Liu.
